# Proposal of a comprehensive definition of modified and other forms of mycotoxins including “masked” mycotoxins

**DOI:** 10.1007/s12550-014-0203-5

**Published:** 2014-06-26

**Authors:** Michael Rychlik, Hans-Ulrich Humpf, Doris Marko, Sven Dänicke, Angela Mally, Franz Berthiller, Horst Klaffke, Nicole Lorenz

**Affiliations:** 1 Chair of Analytical Food Chemistry, Technische Universität München, Alte Akademie 10, 85354 Freising, Germany; 2 BIOANALYTIK Weihenstephan, ZIEL Research Center for Nutrition and Food Sciences, Technische Universität München, Alte Akademie 10, 85354 Freising, Germany; 3 Institute of Food Chemistry, Westfälische Wilhelms-Universität Münster, Corrensstr. 45, 48161 Münster, Germany; 4 Institute of Food Chemistry and Toxicology, University of Vienna, Währingerstr. 38, 1090 Vienna, Austria; 5 Institute for Animal Nutrition, Friedrich-Loeffler-Institue (FLI), Federal Research Institute for Animal Health, Bundesallee 50, Braunschweig, 38116 Germany; 6 Department of Toxicology, University of Würzburg, Versbacher Strasse 9, 97078 Würzburg, Germany; 7 Christian Doppler Laboratory for Mycotoxin Metabolism and Center for Analytical Chemistry, Department IFA-Tulln, University of Natural Resources and Life Sciences, Vienna, Konrad Lorenz Str. 20, 3430 Tulln, Austria; 8 Safety in the Food Chain, Federal Institute for Risk Assessment (BfR), Max-Dohrn-Str. 8-10, 10589 Berlin, Germany

**Keywords:** Definition, Mycotoxins, Masked mycotoxins, Bound mycotoxins, Modified mycotoxins, Hidden mycotoxins, Conjugated mycotoxins, Mycotoxin metabolites, Mycotoxin derivatives

## Abstract

As the term “masked mycotoxins” encompasses only conjugated mycotoxins generated by plants and no other possible forms of mycotoxins and their modifications, we hereby propose for all these forms a systematic definition consisting of four hierarchic levels. The highest level differentiates the free and unmodified forms of mycotoxins from those being matrix-associated and from those being modified in their chemical structure. The following lower levels further differentiate, in particular, “modified mycotoxins” into “biologically modified” and “chemically modified” with all variations of metabolites of the former and dividing the latter into “thermally formed” and “non-thermally formed” ones. To harmonize future scientific wording and subsequent legislation, we suggest that the term “modified mycotoxins” should be used in the future and the term “masked mycotoxins” to be kept for the fraction of biologically modified mycotoxins that were conjugated by plants.

## Background

The rationale behind the new definition presented here was an ongoing discussion about the term “masked mycotoxins.” This term has been introduced as early as 1990 by Gareis et al. to describe a zearalenone glucoside: “Since zearalenone–glycoside is not detected during routine analysis, but hydrolysed during digestion, it seems likely that such ‘masked mycotoxins’ are involved in cases of mycotoxicoses.” Meanwhile, the term “masked mycotoxins” is established internationally, but has been used ambiguously. In 2011, the International Life Science Institute (ILSI) has adopted the following definition: “Mycotoxin derivatives that are undetectable by conventional analytical techniques because their structure has been changed in the plant are designated masked mycotoxins”. According to Berthiller et al. (2013), the term “masked mycotoxins” should exclusively be used for plant metabolites of mycotoxins in order to avoid misunderstandings and confusion.

However, there are other substances derived from mycotoxins, which likewise are not detectable in routine analysis, but which are primarily not produced by plants. Besides mammalian and fungal metabolites these include thermally formed specimens arising in the course of processing. All these compounds are not covered by the term “masked mycotoxins.”

Accordingly, there is a need for a systematical definition of all potential mycotoxin derivatives (beyond the “parent compound”). Therefore, the definition presented here should contribute to the harmonization of terminology in scientific parlance to enable progressing to further questions in the future. From the perspective of consumer health protection, for example, the identification of mycotoxins with all modifications thereof causing toxic effects in humans and animals after primarily oral ingestion is of enormous interest. Accordingly, also data on the bioavailability, toxicology, and analysis of individual mycotoxin derivatives will be discussed in the following.

Although it has been shown in the past few years that glucosides of zearalenone (ZEN-14-Glc) and deoxynivalenol (DON-3-Glc) possess toxic potential, the derivation of toxicological reference values as well as the setting of maximum levels in food and feed is based exclusively on the particular parent compounds (in this case ZEN and DON). The only exceptions so far are (1) the maximum levels of the metabolite aflatoxin M_1_ in milk (Commission Regulation (EC) no. 1881/2006) and (2) a derivation by the Joint FAO/WHO Expert Committee on Food Additives (JECFA) of a provisional maximum tolerable daily intake (PMTDI) as well as an acute reference dose (ARfD) for the group of DON and its two metabolites/precursors 3-acetyl-DON and 15-acetyl-DON (see also chapter “Knowledge in toxicology”). A consideration of other related mycotoxins, e.g., of DON-3-Glc, is currently being controversially discussed. However, the literature data seem to be too limited at the moment for a conclusive decision.

From the perspective of consumer health protection, risk assessment should cover all mycotoxin compounds potentially leading to toxic effects in humans and animals after oral ingestion. In this sense, the exclusive focus on mycotoxin compounds that are produced by plants is insufficient, although Berthiller et al. (2013) argued with a lower toxicity of the latter. However, this cannot be assumed to be a general rule. Therefore, the exclusive use of the term “masked mycotoxins” does not seem to appear adequate. However, since the term “masked mycotoxins” has already been established, it should be used as defined before and limited to plant metabolites of mycotoxins. Further reasons for this recommendation are discussed in the following sections.

## Drawbacks of the term “masked” mycotoxins

As detailed before, “masking” initially referred to analytical issues and no underlying structures or origins were supposed. Since then, the term “masked” has been used ambiguously along with other terms such as “bound,” “hidden,” and “conjugated” until ILSI defined masked mycotoxins as plant metabolites (Berthiller et al. 2013). In the latter review, masked mycotoxins were further differentiated into extractable and bound (non-extractable) mycotoxins and the latter group furthermore into “covalently and non-covalently attached to polymeric carbohydrate or protein matrices.” However, even in the review by Berthiller et al. (2013), non-enzymatically formed mycotoxin modifications such as patulin bound to solids in cloudy apple juices have been termed “masked,” which also underlines the need of a more concise and systematic definition.

Apart from its ambiguous use, there are several further constraints of the term “masked mycotoxins.” In inorganic analysis, for instance, masked is used in a different sense, i.e., as an intended analytical procedure to mask ions in complexometry that usually interfere with the determination of the target ion (Schwarzenbach and Flaschka 1965).

Moreover, as analytical methods rapidly evolve, some compounds (in particular those for which reference substances are available) nowadays might well be “detected during routine analysis” (Gareis et al. 1990) and no longer being masked according to the original definition.

Another weakness of the term “masked” is that the underlying reaction occurring in plants is not intended to “hide” the toxins but simply to detoxify them. Thus “masked” is an anthropocentric term with a negative connotation, which is not justified.

The most striking argument, however, is the missing inclusion of other potentially relevant forms of mycotoxins such as mammalian metabolites or thermally generated modifications.

## New definition proposed

In order to encompass all possible forms in which mycotoxins and their modifications can occur, we hereby propose a systematic definition consisting of four hierarchic levels (Table 1), the highest of which differentiates the free and unmodified forms of mycotoxins from those being matrix-associated and from those being modified in their chemical structure. The following lower levels further differentiate the single specimens in more detail. For a better understanding, Table 1 provides single examples for the different categories. The emphasis of the definition has been placed on processes for generation to include all known and conceivable compounds related to mycotoxins. As some molecules can be generated by different ways, the assignment cannot always be unequivocal, i.e., some compounds belong to more than one category. The rationale behind this is to keep the definition open for yet unknown compounds.

**Table 1:** Systematic definition of “modified mycotoxins”

1st level	2nd level	3rd level	4th level	Example
Free mycotoxins				DON, Aflatoxin B_1_, 3-acetyl-DON, 15-acetyl-DON
Matrix-associated mycotoxins	Complexes, physically dissolved or trapped			
	Covalently bound			Fumonisines bound to starch, OTA- and DON-oligosaccharides
Modified mycotoxins	Biologically modified	Functionalised (phase 1–metabolites)		Aflatoxin B_1_-epoxide
		Conjugated (phase 2 –metabolites)	Conjugated by plants (= masked according to ILSI)	DON-3-glucoside
			Conjugated by animals	DON-3/8/15-glucuronide, HT2-3/4-glucuronide
			Conjugated by fungi	ZEN-14-sulfate
		Differently modified		Deepoxy-DON (=DOM-1)
	Chemically modified	Thermally formed		norDON A-C, N-carboxy-methyl-FB_1_, 14-(R)-OTA
		Non-thermally formed		DON-sulfonate, norDON A-C (under alkaline conditions)

## Definition of the single categories and examples

### Free

According to this definition the term “free” or “unmodified” mycotoxins describes the basic mycotoxin structures formed as toxic secondary metabolites by various fungi in well-known biosynthetic pathways. Examples are ochratoxin A, aflatoxin B_1_, fumonisin FB_1_, zearalenone, deoxynivalenol, and also 3- and 15-acetyl-deoxynivalenol, which are also formed during the biosynthetic pathway. But especially the latter two substances, 3- and 15-acetyl-deoxynivalenol, may be placed into more than one category. Recent developments of transgenic plants with trichothecene-O-acetylase activity for conjugation of DON may render 3-acetyl-DON also to fall into the category conjugated by plants (masked) (Karlovsky 2011).

### Matrix-associated

This term should be used for mycotoxins, which (1) form either complexes with matrix compounds or are physically dissolved or trapped or (2) are covalently bound to matrix components or a combination of both effects. Examples for covalently bound forms of mycotoxins are fumonisins bound to starch or proteins as was proposed and shown for the first time by Shier et al. (2000) using radiolabelled fumonisin B_1_ in model experiments although the exact structure was not clear (Shier 2000). The covalent binding of fumonisins to starch and proteins was later confirmed by Seefelder et al. (2003) in model experiments using methyl α-D-glucopyranoside as starch model and various amino acids as protein model. Detailed MS and NMR studies showed that fumonisins are able to bind to polysaccharides and proteins via their two tricarballylic acid side chains (Seefelder et al. 2003). Recently also the covalent binding of ochratoxin A (OTA) to polysaccharides via the carboxylic acid group of OTA during the roasting of coffee has been shown by Bittner et al. (2013). Also, DON-oligosaccharides have been described recently (Zachariasova et al. 2012).

### Modified

The term “modified mycotoxins” describes any modification of the basic chemical structure of mycotoxins either by chemical or biological modifications.

#### Biologically modified

Biologically modified mycotoxins include on the third level (see Table 1) any functionalization during phase 1-metabolism, for example aflatoxin B_1_ exo-8,9-epoxide, which is the aflatoxin metabolite that reacts covalently with DNA to form the adducts responsible for the toxic effects.

Furthermore, mycotoxin conjugates such as phase 2-metabolites are also defined as biologically modified. These includes on the 4th level (1) conjugation reactions by plants such as the formation of DON-3-Glc or ZEN-14-Glc, which are defined as masked mycotoxins by ILSI (Berthiller et al. 2013); (2) conjugation reactions by animals such as the formation of DON-3/8/15-glucuronides or HT2-3/4-glucuronides, with the structures of new glucuronides recently having been elucidated (Welsch and Humpf 2012; Uhlig et al. 2013); and (3) conjugations by fungi as for example the formation of ZEN-14-sulfate (Plasencia and Mirocha 1991) or the formation of N-acyl and O-acyl fumonisins by *Fusarium verticillioides* (Bartók et al. 2013). All other biological modifications are summarized under the term “differently modified” (see Table 1) and include for example deepoxy-DON (DOM-1) as an intestinal metabolite of DON, which is formed by the microbiota of animals and humans (Eriksen et al. 2002; Gratz et al. 2013).

#### Chemically modified

Chemically modified mycotoxins are currently the largest group of modified mycotoxins and can be classified as “thermally formed” and “non-thermally formed” on the third level (see Table 1).

Thermal degradation reactions as well as thermal modifications occur during food and feed processing including baking, roasting, frying, or extruding. Thermal degradation products have been described for several mycotoxins. A prominent example is fumonisin FB_1_ which can react in a Maillard-type reaction with reducing sugars leading to N-(1-deoxy-D-fructos-1-yl) fumonisin B_1_ and *N*-(carboxymethyl)fumonisin B_1_ (for a review, see Humpf and Voss 2004). The latter one is the stable end product and detectable in processed corn samples in concentrations up to 76 μg/kg (Seefelder et al. 2001).

Thermal degradation products of DON have been identified as norDON A-F and 9-hydroxymethyl DON lactone (Bretz et al. 2006; Young et al. 1986) in model heating experiments. However, some of these compounds are also formed under alkaline conditions without thermal treatment. The significance of these DON degradation products was proven by analyzing commercially available food samples. Only the three degradation products norDON A, B, and C were detectable in 29–66 % of the samples with mean concentrations ranging from 3 to 15 μg/kg. Further examples of thermal modifications of mycotoxins are norNIV A, norNIV B, norNIV C, and NIV lactone as degradation products of nivalenol of which only norNIV B was detectable in commercially available samples (Bretz et al. 2005).

As degradation reactions of ochratoxin A during the roasting of coffee, the isomerization to 14-(R)-ochratoxin A and the decarboxylation to 14-decarboxy-ochratoxin A were identified as main reactions. The analysis of 15 coffee samples from the German market revealed that the ochratoxin A diastereomer 14-(R)-ochratoxin A was formed in amounts of up to 25.6 % relative to ochratoxin A and the decarboxylation product was only detectable in traces(Cramer et al. 2008).

Model experiments with T-2 toxin revealed three thermal degradation products of which only one was detectable in traces in commercial food samples (Beyer et al. 2009).

Examples for non-thermal modifications of mycotoxins are the formation of hydrolyzed fumonisins (HFB_x_) or the above mentioned norDON A–C degradation products formed under alkaline conditions (Humpf and Voss 2004; Young et al. 1986). Other examples are degradation reactions induced by UV light as was recently shown for ochratoxin A and citrinin (Schmidt-Heydt et al. 2012) or DON sulfonate generated by treatment of contaminated feed with sodium bisulfite (Beyer et al. 2010).

At this point, we would like to emphasize once more that some compounds fall in different categories. For example, hydrolyzed fumonisins (HFB_x_), on the one hand, are biologically modified and are formed by the intestinal microbiota (Fodor et al. 2008) and, on the other hand, are formed under alkaline conditions during food processing (Humpf and Voss 2004).

## Analytical considerations

In order to collect occurrence data and to assess the effects of consumption of mycotoxins on the health of animals and humans, a reliable analytical detection not only of free but also of modified and matrix-associated mycotoxins is inevitable. For the determination of free mycotoxins in food and feed, a huge variety of analytical methods has already been developed and mainly based either on chromatographic methods such as thin layer chromatography, gas chromatography, or liquid chromatography (also coupled to mass spectrometry) or on immunochemical methods such as enzyme-linked immunosorbent assays (ELISA). In contrast to chromatographic methods, immunochemical methods sometimes respond to more than one compound (i.e., the free mycotoxin and its modification) depending on the antibody used for analysis and resulting in an undistinguishable signal for more than one compound (Goryacheva et al. 2009; Goryacheva and De Saeger 2012). This effect is known as “cross-reactivity” and was shown for example for DON and DON-3-Glc (Ruprich and Ostry 2008). In this case, cross-reactivity varies depending on the antibody between 8 and 157 % (Tangni et al. 2010). Another example is the thermal isomerization product 14-(R)-ochratoxin A, which is only binding to immunoaffinity columns of one supplier, although only one stereocenter is different compared to OTA (Cramer et al. 2008).

In general, there are three different approaches for the analysis of modified mycotoxins: direct and indirect determination as well as not-targeted analysis.

Direct analysis offers the advantage that conventional, standardized analytical methods are suitable, but these need to be adjusted and optimized according to the structurally related chemical properties (polarity, altered extraction behavior, detection characteristics, etc.) compared to the free mycotoxins. The huge disadvantage of direct analysis is the fact that up to now only a few modified mycotoxins (e.g., DON-3-Glc) are available as reference substances for analysis.

This problem can be circumvented by indirect determination, where the modified forms are transformed into the native mycotoxin, which then can be analyzed by “routine analysis.” Therefore, chemical and enzymatic hydrolysis (e.g., for conjugates or bound compounds), reduction (e.g., for biotransformation products of the phase I reaction) and other specific reactions are used during sample preparation. However, indirect analysis of modified mycotoxins provides only limited information about the structure and amount of the mycotoxin specimens originally present in the sample. Thus, a distinguished estimation of the deduced toxicological effects in humans or animals after consumption of food/feed contaminated with native and modified mycotoxins are hardly possible.

So, it can be concluded that in the last years, more and more direct methods based on LC-MS have been developed and up to now, have become the methods of choice for the determination of modified mycotoxins (Berthiller et al. 2007; Li et al. 2013). More detailed information on the analysis of modified mycotoxins can be found in recent publications (Dall’Asta et al. 2010; Di Mavungu and De Saeger 2011; De Boevre et al. 2012; Berthiller et al. 2013).

However, it is not only possible to identify and quantify known substances, the so-called target mycotoxins. Also, previously unknown derivatives of mycotoxins can be detected by so-called non-targeted analysis using, for example, high-resolution mass spectrometry (HR-MS) coupled with liquid chromatography like UPLC (Cirlini et al. 2012). In this way, Nakagawa et al. (2013) identified new modified mycotoxins (mycotoxin glucosides) derived from type A trichothecenes in commercially available corn powder reference material. These new glucosides (neosolaniol–glucoside and diacetoxyscirpenol–glucoside) were identified based on accurate mass measurements of characteristic ions and fragmentation patterns using a high-resolution liquid chromatography–Orbitrap mass spectrometric analysis (Nakagawa et al. 2013). A review of MS approaches to study phase II metabolites in general has been published (Levsen et al. 2005).

Overall, all analytical methods serve the purpose to obtain differentiated data of free and modified mycotoxins in food/feed, to elucidate the occurrence and exposition with these compounds.

## Occurrence

Long before the term “masked mycotoxins” emerged, modified mycotoxins have been discovered. For example, aflatoxin M_1_ as a metabolite of aflatoxin B_1_ excreted with milk has been reported almost 50 years ago (Masri et al. 1967).

In the last years, increasing quantitative data of modified mycotoxins have been published, in particular in the field of *Fusarium* toxins. This is obviously due to emerging awareness and the predominance of LC-MS/MS equipment in laboratories dealing with mycotoxins.

Among all modified mycotoxins, most occurrence data exist for DON-3-Glc, which was detected in naturally contaminated maize and wheat for the first time in 2005 (Berthiller et al. 2005). Subsequent surveys indicated sporadically high contaminations of DON-3-Glc exceeding 1,000 μg/kg in naturally contaminated wheat (Berthiller et al. 2009). Molar ratios of DON-3-Glc in relation to unmodified DON were found to be highly variable in the range between 20 and over 70 % depending on cereal species and genotype, country and year of harvest (Berthiller et al. 2009; Desmarchelier and Seefelder 2011; De Boevre et al. 2012). Apart from wheat and maize, DON-3-Glc also has been detected in oats (De Boevre et al. 2012) and barley (Lancova et al. 2008) thus being transferred into beer made of the latter (Kostelanska et al. 2009). Cereal contamination with DON-3-Glc was reported to occur worldwide according to surveys from the UK (Vendl et al. 2010), the Czech Republic (Malachova et al. 2011), China (Li et al. 2012), and Canada (Tran et al. 2011). For occurrence of further modified mycotoxins, e.g., conjugates of ZEA (De Boevre et al. 2012 and 2013; Berthiller et al. 2009) or of T-2 and HT-2 toxins (Veprikova et al. 2012) or modified fumonisins (Humpf and Voss 2004; Falavigna et al. 2012), some market reviews have been published, but the reported data are still rather limited.

## Knowledge in toxicology

Potential exposure to modified mycotoxins due to their presence in food and feed raises concern that modified mycotoxins may pose an additional risk to human and animal health. While conjugated and matrix-associated mycotoxins may be cleaved by the gut microflora (e.g., DON-3-Glc → DON, Nagl et al. 2012) or endogenous digestive enzymes (e.g., fumonisins bound to starch → fumonisins, Humpf and Voss 2004) to the parent compound and thus add to the systemic exposure and toxicity of the free mycotoxin, other modified mycotoxins may be less, equally or even more toxic than their parent compound. To understand the toxicological relevance and contribution of modified mycotoxins to the overall health risk resulting from dietary intake of mycotoxins, it is thus critical to assess the bioavailability and toxic potential of modified mycotoxins. In particular, while available in vitro studies mostly suggest lower cytotoxic potential of modified mycotoxins compared to their parent compound as exemplified by DON derivatives in Table 2, few in vivo data on the absorption, distribution, metabolism, and excretion (ADME) and toxicity of modified mycotoxins are yet available.

**Table 2:** Cytotoxicity of modified DON derivatives compared to DON in different cell culture systems in vitro

Level of hierarchy	Modified DON	Cell culture system	Assay	IC_50_ (μM)		Reference
				Modified DON	DON	
1st Level: free mycotoxins	15-acetyl-DON	3T3 cells	BrdU	1.5	1.5	Eriksen et al. 2004
		Bovine PBMC	MTT	0.5	0.5	Dänicke et al. 2011
	3-acetyl-DON	3T3 cells	BrdU	14.5	1.5	Eriksen et al. 2004
		Bovine PBMC	MTT	2.6	0.5	Dänicke et al. 2011
1st Level: modified mycotoxins						
2nd Level: biologically modified						
3rd Level: conjugated (phase II metabolites)						
4th Level: conjugated by plants						
	DON-3-O-glucoside	WG-T/T	LA	(>20)^b^	0.8^a^	Poppenberger et al. 2003
4th Level: conjugated by animals						
	DON-3-glucuronide	K562	MTS	(>270)^b^	1.31	Wu et al. 2007
3rd Level: differently modified						
	DOM-1	3T3 cells	BrdU	83.1	1.5	Eriksen et al. 2004
		Bovine PBMC	MTT	(>18)^b^	0.5	Dänicke et al. 2011
		Porcine PBMC	MTT	(>23)^b^	1.18	Dänicke et al. 2010
		IPEC-1	MTT	(>23)^b^	1.33	Dänicke et al. 2010
		IPEC-J2	MTT	(>23)^b^	2.97	Dänicke et al. 2010
2nd Level: chemically modified						
3rd Level: thermally formed						
	nor-DON-A	IHKE	WST-8	(>100)^b^	1.1	Bretz et al. 2006
3rd Level: non-thermally formed						
	DON-sulfonate	Porcine PBMC	MTT	(>17)^b^	1.18	Dänicke et al. 2010
		IPEC-1	MTT	(>17)^b^	1.33	Dänicke et al. 2010
		IPEC-J2	MTT	(>17)^b^	2.97	Dänicke et al. 2010
		HepG2	MTT	(>100)^b^	41.0	Beyer 2009
		Caco-2	MTT	9.2	2.0	Beyer 2009
		IHKE	MTT	8.1	1.6	Beyer 2009

*3T3 cells* Swiss mouse fibroblasts; *BrdU* 5-bromo-2-deoxyuridine, DNA synthesis; *Caco*-*2* a human epithelial colorectal adenocarcinoma cell line; *HepG2* a human hepatoma cell line; *IHKE* an immortalized human kidney epithelial cell line; *IPEC*-*1 and IPEC*-*J2* non-transformed Intestinal Porcine Epithelial Cell lines; *K562* erythroleukemia cell line; *MTS* methylthiazol tetrazolium, cell viability; *MTT* (3-[4,5-dimethylthiazol-2-yl]-2,5-diphenyl-tetrazolium bromide), cell viability; *PBMC* primary peripheral blood mononuclear cells; *WG*-*T*/*T* wheat germ extract-based coupled transcription/translation system; *WST-8* [2-(2-methoxy-4-nitrophenyl)-3-(4-nitrophenyl)-5-(2,4-disulfophenyl)-2H-tetrazolium, monosodium salt], cell viability

^a^Linearly interpolated from published data

^b^No IC_50_ derivable, highest tested concentrations are given in brackets

The best studied examples so far involve derivatives of the *Fusarium* mycotoxins DON and ZEN, e.g., DON-3-Glc, the acetyl derivatives 3-acetyl-deoxynivalenol (3-Ac-DON), and 15-acetyl-deoxynivalenol (15-Ac-DON), and ZEN-14-Glc.

In vitro studies on the comparative toxicity of DON and its acetylated derivatives demonstrate that 15-Ac-DON and 3-Ac-DON are equally or less toxic compared to DON (Table 2). In contrast to this and even more important, studies on the acute oral toxicity of acetylated DON derivatives in mice revealed similar LD_50_ values for DON and its 3-acetyl and 15-acetyl metabolites, demonstrating that the oral toxicities of 3-Ac-DON and 15-Ac-DON in vivo are comparable to that of DON (Forsell et al. 1987; Yoshizawa and Morooka 1974). In evaluating the risk resulting from DON exposure, the Joint FAO/WHO Expert Committee on Food Additives (JECFA) considered the acetylated DON derivatives to contribute to the total DON-induced toxicity and established a group PMTDI and group ARfD for DON and its acetylated derivatives (JECFA 2011). Similar to Ac-DONs, DON-3-Glc may, at least in part, be hydrolyzed to DON in the gastrointestinal tract and thus may contribute to the total dietary exposure to DON. Following oral administration of DON-3-Glc to rats, DON and its metabolites deoxynivalenol-glucuronide (DON-3-GlcA) and deepoxy deoxynivalenol (DOM-1) were recently identified as urinary metabolites of DON-3-Glc (Nagl et al. 2012). Studies on the hydrolytic fate of DON-3-Glc during digestion indicate that DON-3-Glc may be cleaved by intestinal bacteria and thus become bioavailable as DON (Berthiller et al. 2011). Therefore, it may be concluded that data on the toxic potential of DON-3-Glc using cellular or molecular endpoints in vitro such as inhibition of protein synthesis (Poppenberger et al. 2003) may not be accurate predictors of systemic toxicity of DON-3-Glc in whole animals or humans.

DOM-1 is another example for cytotoxic assays being insufficient to evaluate the overall toxicological relevance. Although DOM-1 seems to be generally low toxic both for bovine and porcine peripheral blood mononuclear cells (PBMC) (Table 2) the interspecies differences in pre-absorptive modification capability need to be considered in evaluating the overall toxicity of DON for cattle and pigs. While DON is nearly completely modified to DOM-1 in cattle by rumen micro-organisms prior systemic absorption this modification occurs also in the hindgut of pigs, but only very small amounts of non-absorbed free DON reach this intestinal segment. Therefore, in DON-exposed cattle, mostly DOM-1 is detectable in blood while in pigs the majority is in the form of free DON (e.g., Dänicke and Brezina 2013).

Similarly to DON-3-Glc, glycosylation of the estrogenic mycotoxin ZEN by plant UDP-glucosyltransferases to its derivative ZEN-14-Glc has been shown to prevent binding to estrogen receptors. While conjugation may present a means of detoxification to protect the plant (Poppenberger et al. 2006), it is important for human and animal health risk assessment to consider that ZEN-14-Glc may be cleaved during digestion and release its active parent compound as demonstrated in pigs (Gareis et al. 1990).

These examples emphasize the need for toxicokinetic and toxicity data on modified mycotoxins to allow human and animal health risk assessment. They also highlight some of the issues that need to be considered:

- The systemic and local toxicological effects of modified mycotoxins depend on their release from the matrix, metabolic modification within the digestive tract, absorption, biotransformation, and finally, the toxicodynamic potencies of modified mycotoxins and their metabolites. Co-exposure to free and modified mycotoxins needs to be considered in evaluating the overall health risks
- Assessment of the comparative toxicity of modified mycotoxins in isolated cells may be a poor predictor of toxicity in vivo as it rarely considers metabolic conversion to the active metabolite
- Besides systemic toxicity, potential local effects of modified mycotoxins (or their metabolites) on the gastrointestinal tract need to be considered
- Significant species differences and intra-species variation in the composition and activity of the gut microflora and thus metabolic conversion and bioavailability of toxicologically active metabolites may lead to significant inter- and intra-species differences in the toxicity of modified mycotoxins

## Implications for legislation

The need for regulating modified mycotoxins has been recognized by European regulatory bodies, but due to the lack of toxicological data, implementation remained vague until now. More specifically, the European Commission (EC) asked the European Food Safety Authority (EFSA) in July 2013 for a scientific opinion on the risks for animal and public health related to the presence of DON, metabolites of DON, and masked DON in food and feed (M-2013-0260). EFSA accepted the mandate (EFSA-Q-2013-00721) and an opinion is expected in the near future (www.efsa.europa.eu). As mentioned before, only the acetylated derivatives of DON have been added to DON for a PMTDI and an ARfD of the DON group for sample-specific risk assessments (JECFA 2011).

Furthermore, EFSA received a request from EC for a scientific opinion on the risks for animal and public health related to the presence of metabolites and masked or bound forms of certain mycotoxins in food and feed (EFSA-Q-2013-00720).

Moreover, the European Commission pointed to the need to “analyse also the masked mycotoxins in particular the mono- and di-glycosylated conjugates of T-2 and HT-2 toxin” in the current recommendation on indicative levels for the sum of T-2 and HT-2 toxins in cereals and cereal-based foods (European Commission 2013).

For all reasons mentioned before and in particular to harmonize future scientific wording and subsequent legislation, we suggest that the term “modified mycotoxins” should be used in future. As the term “masked mycotoxins” has been already introduced for plant metabolites, we propose this term to be kept for the fraction of biologically modified mycotoxins, conjugated by plants (Table 1).
